# Facile method for delivering chikungunya viral replicons into mosquitoes and mammalian cells

**DOI:** 10.1038/s41598-021-91830-y

**Published:** 2021-06-10

**Authors:** Hui-Chung Lin, Der-Jiang Chiao, Chang-Chi Lin, Szu-Cheng Kuo

**Affiliations:** 1 Graduate Institute of Medical Sciences, National Defense Medical Center, Taipei, 11490 Taiwan; 2 Institute of Preventive Medicine, National Defense Medical Center, Taipei, 11490 Taiwan; 3 Department and Graduate Institute of Microbiology and Immunology, National Defense Medical Center, Taipei, 11490 Taiwan

**Keywords:** Biotechnology, Microbiology, Medical research

## Abstract

Reverse genetics is an important tool in the elucidation of viral replication and the development of countermeasures; however, these methods are impeded by laborious and inefficient replicon delivery methods. This paper demonstrates the use of a baculovirus to facilitate the efficient delivery of autonomous CHIKV replicons into mosquito and mammalian cells in vitro as well as adult mosquitoes in vivo. The efficacy of this approach was verified via co-localization among an eGFP reporter, nsP1, and dsRNA as well as through the inhibition of an RNA-dependent RNA polymerase (RdRp) null mutation (DDAA) in nsP4, or the treatment of a known antiviral compound (6-azauridine). We also investigated the correlation between CHIKV replicon-launched eGFP expression and the effectiveness of CHIKV replicon variants in inducing IFN-β expression in human cell lines. This delivery method based on a single vector is applicable to mosquito and mammalian cells in seeking to decipher the mechanisms underlying CHIKV replication, elucidate virus–host interactions, and develop antivirals. This study presents an effective alternative to overcome many of the technological issues related to the study and utilization of autonomous arbovirus replicons.

## Introduction

Chikungunya virus (CHIKV) is an old-world alphavirus of the Togaviridae family grouped genetically within the Semliki Forest virus complex^1^, which is transmitted by *Aedes* mosquitoes. CHIKV infection generally causes fever, joint pain, skin rash, and arthralgia, and is in some cases fatal^2^. In the 1950s, human cases were first reported in Africa, with *Aedes* mosquitoes identified as the primary vector of the sylvatic cycle^3,4^. Following a subsequent outbreak in 2004, researchers determined that the virus had expanded into novel ecological niches^5^. Rising global temperatures and wide-scale transportation are raising further concerns about the spread of CHIKV, as there are at present no effective vaccines or drugs for treating the infection^5,6^. CHIKV is an enveloped virus measuring roughly 70 nm in diameter with an 11.8 kb single-stranded, positive-sense RNA genome^1^. The genome of CHIKV comprises a 5′ cap untranslated region (UTR), followed by two open reading frames (ORF) encoded four non-structural proteins (nsP1–4), five structural proteins (C–E3–E2–6K–E1), and a 3′ terminal poly-A tail^7^. Like other alphaviruses, the polyproteins of four non-structural proteins (nsP1–4) of CHIKV comprise the viral replication machine^1,8^. Processing non-structural polyproteins to form the nsP1/P23/nsP4 replicase complex results in the production of genomic and subgenomic RNAs^9^. Subgenomic mRNA transcribed from the subgenomic promoter (accumulating to 10^6^ molecules per cell) is involved in the translation of structural proteins and virion assemble. CHIKV is transmitted from mosquitoes into mammals; however, the characteristics of the alphavirus infection differ in the two hosts. Alphavirus growth in mosquito cells is characterized by non-cytopathic effect (CPE) and persistency^10^. Conversely, alphavirus proliferation in vertebrate cells quickly induces host shut-off and CPE related to viral proteins^11^. The characteristics of alphavirus infection can be attributed to interactions among viral and cellular factors^9^. Among the viral factors, RdRp mutant (DDAA) in nsP4 prevents CHIKV replicons from replicating (in both mammalian and mosquito cells), whereas G1332V mutation in the nsP2 can lead to a 60-fold increase in replication (in C6/36 cells *in trans*)^12^. nsP2 mutations in mammalian cells are also associated with the attenuation of type I interferon expression^13–16^. Unraveling the mechanisms underlying host-virus interactions could conceivably be of value in the development of countermeasures aimed at blocking alphavirus transmission. Autonomously replicating replicons lacking the structural proteins necessary for viral particle formation are often replaced with a reporter gene (e.g., eGFP). Viral replicons capable of recapitulating various steps of the replication cycle without using a live virus could increase accessibility. Transfection of DNA- and RNA-based alphavirus replicons is commonly used to investigate viral replication and assess the efficacy of vaccines and antivirals^12,17–23^. 6-azauridine (6-AU) has been used as an antiviral against CHIKV infection to evaluate the expression of CHIKV replicon-mediated reporter gene^23^. Virus-based alphavirus replicons have also been established to improve the efficiency of replicon delivery into mammalian cells^24–26^. At present, we understand far less about mosquito-alphavirus interactions than vertebrate-alphavirus interactions, due largely to the low efficiency of nucleic acid-based replicon delivery^27^ and a lack of suitable virus-based replicon systems. The recombinant baculovirus (Bac-CMV/SFV-EGFP) developed by Pan et al. exhibited pronounced SFV replicon-launched eGFP expression in mammalian cells^25^. The human cytomegalovirus (CMV) promoter has been extensively utilized for transgene expressions in mammalian cells, and has been used as a functional shuttle allowing the rescue of infectious clones of West Nile virus from C6/36 cell^28^. Previous studies have demonstrated the efficiency of using baculovirus to deliver heterologous genes into mosquito cells (BacMos)^29^. In the current study, we demonstrated the use of a baculovirus for the efficient delivery of autonomous CHIKV replicons into the mosquito and mammalian cells in vitro (hereafter referred to as dual-host cells) as well as adult mosquitoes in vivo.

## Results

### In vitro functional analysis

**Figure 1:**
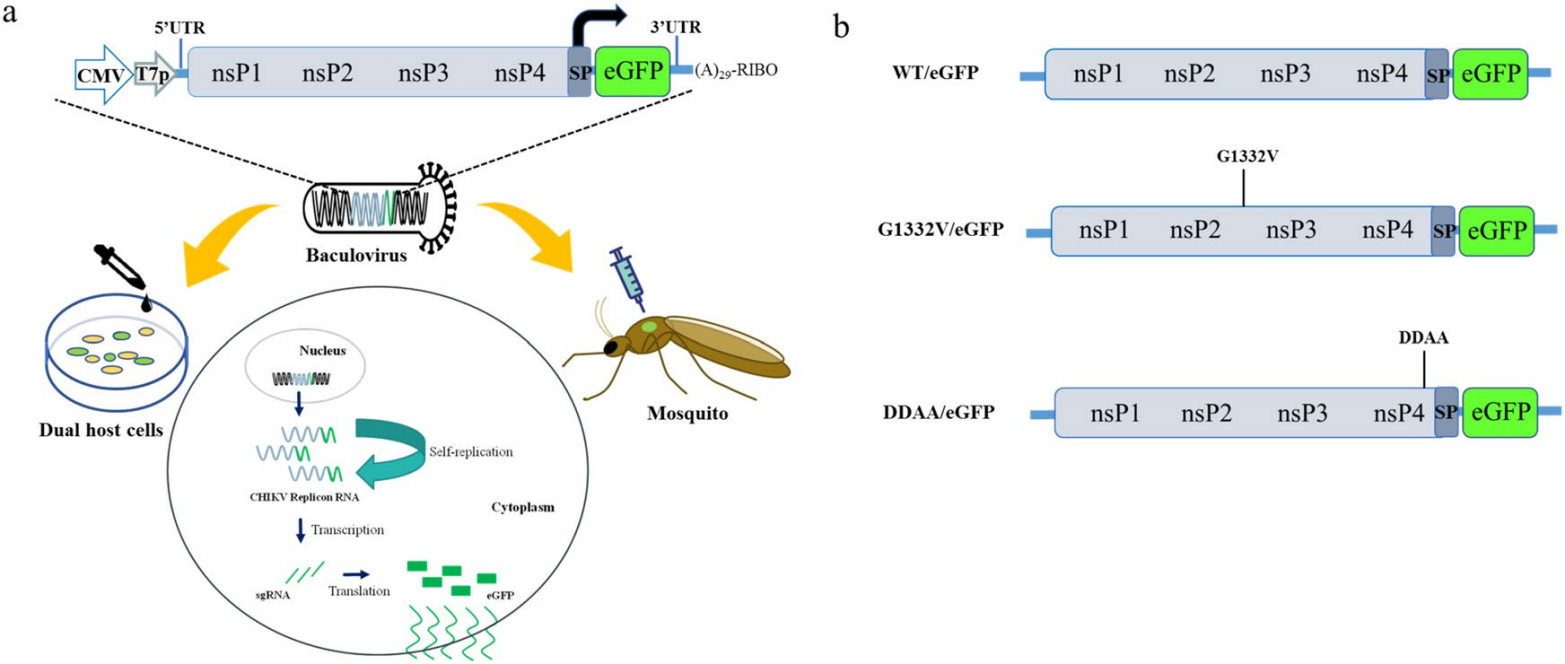
Baculovirus vector used to shuttle CHIKV replicons into mosquito and mammal cells: (**a**) Schematic illustration showing CHIKV replication cycle in mosquito and mammal cells using a single baculovirus. A recombinant baculovirus bearing a DNA cassette of CHIKV replicon-eGFP under the control of a CMV promoter. The baculovirus efficiently delivered CHIKV replicon-eGFP DNA into the nuclei of mosquitoes and mammal cells and adult mosquitoes to induce the expression of eGFP via transduction. T7p, T7 polymerase promoter; 5′ UTR, 5′ untranslated region of CHIKV genome; nsP1–4, non-structural proteins 1–4; SP, subgenomic promoter; 3′ UTR, 3′ untranslated region of CHIKV genome; (A)_29,_ 29 A residues; RIBO, ribozyme site; sgRNA, subgenomic RNA. (**b**) Schematic diagram showing CHIKV replicon/eGFP constructs. WT/eGFP is a CHIKV replicon derived from LR2006_OPY1strain; G1332V/eGFP, WT/eGFP mutated penultimate glycine residue (P2 residue of 2/3 site) to valine of nsP2; DDAA/eGFP, WT/eGFP mutated the polymerase active site motif Gly-Asp-Asp to Gly-Ala-Ala of nsP4.

**Figure 2:**
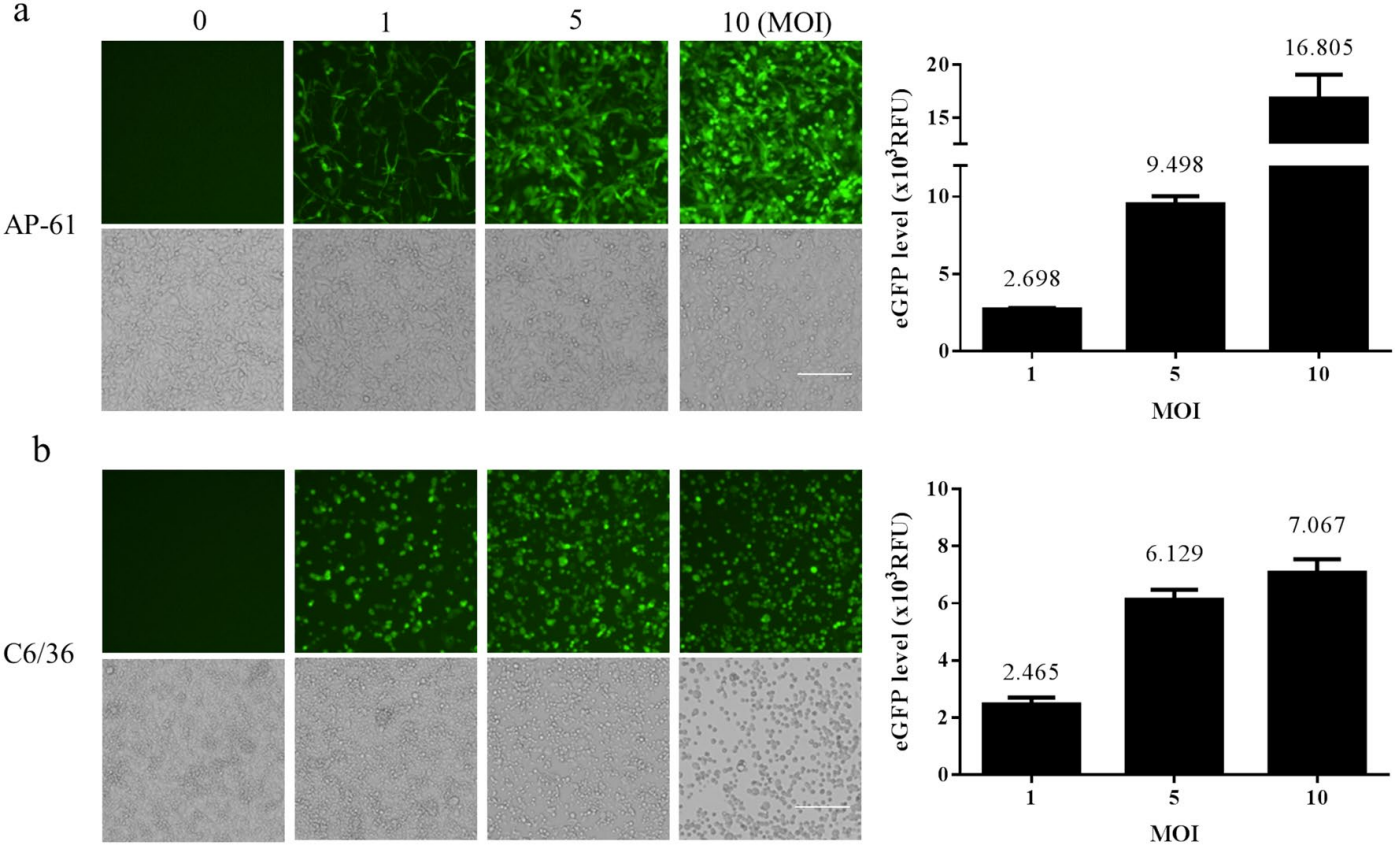
Autonomous CHIKV replicon-mediated expression of eGFP in baculovirus transduced into mosquito cell lines, AP-61 (**a**) or C6/36; (**b**) cells were transduced using WT/eGFP at indicated MOIs of 0, 1, 5 or 10 in triplicate. At 2 dpt, eGFP-expressing cells were photographed using green (upper panels) or bright field (lower panels) filters. Expression levels of eGFP were quantified (right part). Error bars represent the standard deviation.

**Figure 3:**
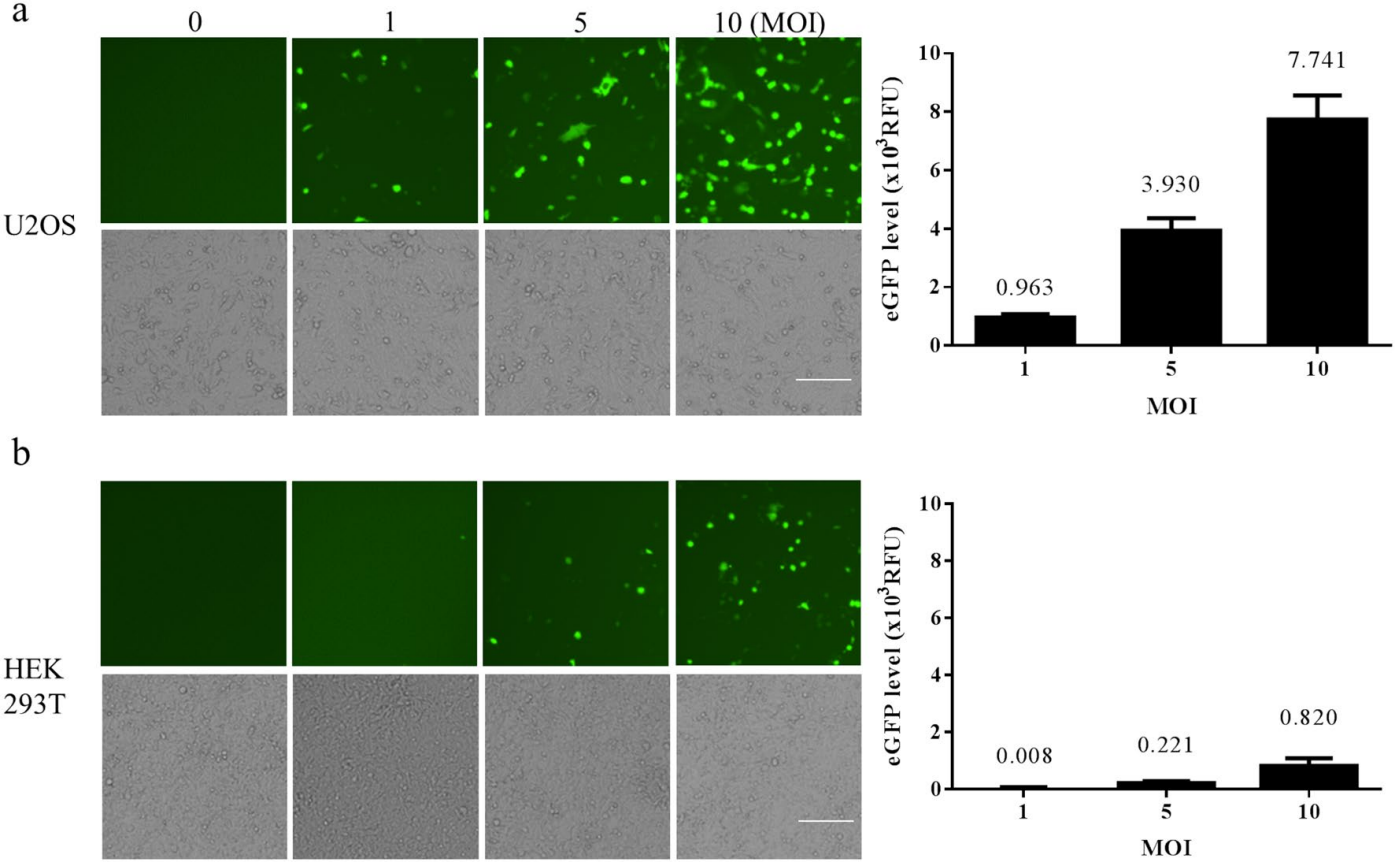
Autonomous CHIKV replicon-mediated expression of eGFP via baculovirus transduction in mammalian cell lines, U2OS (**a**) or HEK293T (**b**) cells via transduction with WT/eGFP at indicated MOIs of 0, 1, 5 or 10 in triplicate. At 2 dpt, eGFP-expressing cells were photographed using green (upper panels) or bright field (lower panels) filters. Expression levels of eGFP were quantified (right part). Error bars represent the standard deviation.

**Figure 4:**
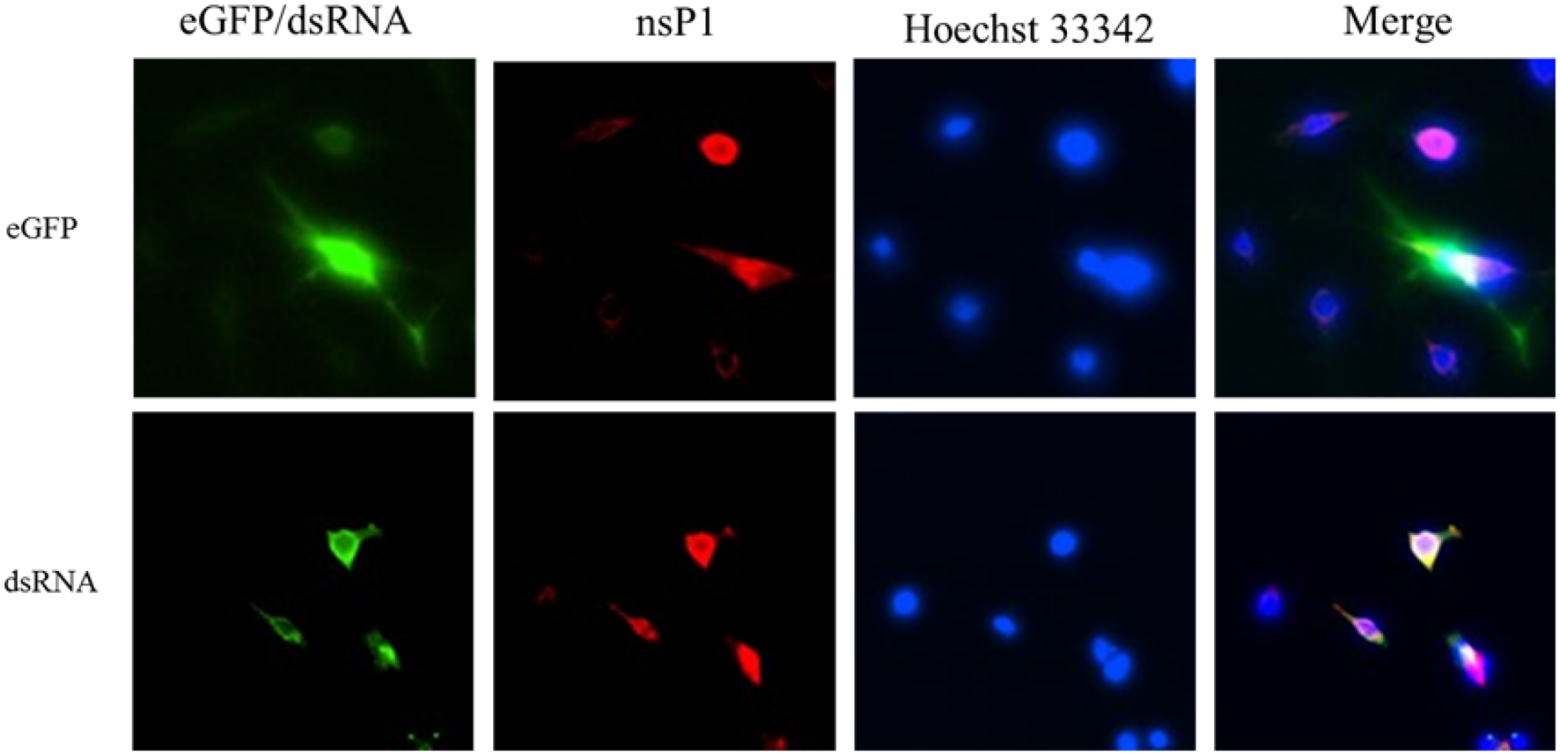
Characterization of autonomous CHIKV replicon using IFA. AP-61cells were transduced with W/eGFP at MOI of 0.5. At 4 dpt, cells were fixed and co-stained with Mab anti-eGFP antibodies, rabbit anti-CHIKV nsP1 serum, and Hoechst 33342 (upper panels), or co-stained with Mab anti-dsRNA, rabbit anti-CHIKV nsP1 serum, and Hoechst 33342 (lower panels). Merged figures of the three are displayed in the right panels.

We established a method for the efficient delivery of autonomous CHIKV replicons into mosquito and mammalian cells in order to assess the self-replicating CHIKV replicon-mediated expression of eGFP (Fig. 1a). Recombinant baculovirus bears a CMV promoter-directed wild type (WT/eGFP) or CHIKV replicon variant (G1332V/eGFP and DDAA/eGFP) containing the eGFP gene under the transcription control of CHIKV 26S subgenomic promoter (Fig. 1b). To determine whether the CHIKV replicon was functional, two mammalian cells (U2OS and HEK293T) and two mosquito cells (AP61 and C6/36) were serially transduced with WT/eGFP. The results (Figs. 2, 3) indicate the strong dose-dependency of eGFP expression in mosquito and mammalian cells, which was particularly pronounced in mosquito cells. Note that eGFP levels obtained from mosquito cells should far exceed those obtained from mammalian cells. The most pronounced eGFP expression was detected in WT/eGFP-transduced AP-61 cells. All transduced cells except C6/36 cells presented eGFP expression with dose-dependency proportional to the MOI. Note that at MOIs of 5 and 10, WT/eGFP-transduced C6/36 cells presented a rounded morphology; however, at an MOI of 10, WT/eGFP-transduced AP-61 cells appeared normal. This is an indication that C6/36 cell are perhaps more sensitive to WT/eGFP transduction than are AP-61 cell. Next, to examine the correlation of eGFP expression and CHIKV replication, immunofluorescence assay (IFA) was conduct to detect eGFP, dsRNA (intermediate of CHIKV replication) and nsP1(viral protein of CHIKV) in transduced AP-61 cells. We also observed the specific co-localization of eGFP, dsRNA and nsP1 in WT/eGFP-transduced AP-61 cells (Fig. 4), indicating RNA replication. To confirm the identity of the autonomous CHIK replicon, we performed functional analysis of IVT-RNA from WT/eGFP. The results (Fig. S1) revealed a high eGFP-positive ratio in IVT-RNA-electroporated BHK-21 cells, indicating the successful expression of eGFP by self-amplified RNA transcripted from the transfer vector of WT/eGFP. Taken together. these results revealed that the autonomous CHIKV replicon is easily delivered into mosquito as well as mammalian cells using a single baculovirus vector.

### Evaluation of antiviral targeting CHIKV replicon

**Figure 5:**
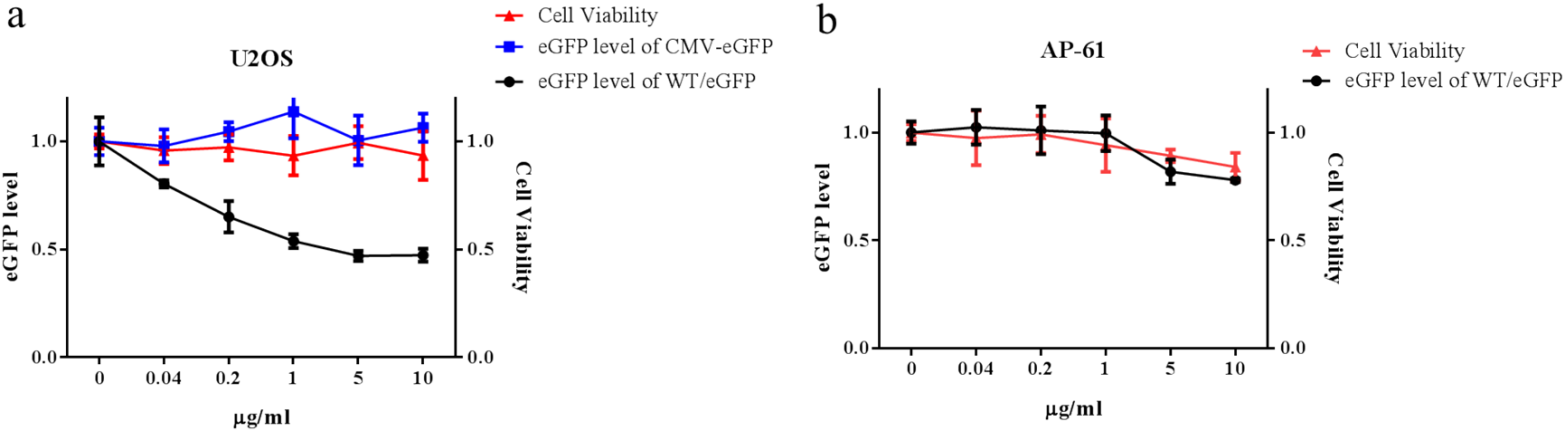
Effect of 6-azaudine in inhibiting CHIKV replicon-mediated expression of eGFP in dual host cell lines. U2OS cells (**a**) were transduced in triplicate using either WT/eGFP or CMV-GFP as a negative control, respectively at an MOI of 5 or 1. AP-61 cells (**b**) were transduced in triplicate using WT/eGFP at an MOI of 1. Mock or transduced cells growth within a range of 6-AU (0–10 μg/ml) over a period of 24 h. eGFP expression and cell viability were quantified using eGFP or MTT. Error bars indicate standard deviation. Data were combined from three independent experiments.

In order to confirm the correlation of eGFP expression and CHIKV replication, we assessed the efficacy of this proposed system in antiviral assays by examining the inhibition effect of 6-AU on CHIKV replicon-launched eGFP expression. The eGFP expression was assessed after culturing WT/eGFP-transduced AP-61 cells as well as WT/eGFP- or CMV-GFP-transduced U2OS cells (as a control) in the presence of 6-AU at specified dilutions. As shown in Fig. 5a, 6-AU treatment reduced eGFP expression in WT/eGFP-transduced U2OS cells in a dose-dependent manner; however, it had no effect on eGFP expression in CMV-GFP-transduced U2OS cells. We also calculated the 50% inhibitory concentration (IC50) and 50% cytotoxicity concentration (CC50) of 6-AU in U2OS cells at concentrations of 1 μg/ml and > 10 μg/ml. Interestingly 6-AU was shown not to affect CHIKV replicon-launched eGFP expression in AP-61 cells (Fig. 5b). Overall, 6-AU exhibited a mammalian cell-biased inhibitory effect on baculovirus-mediated CHIKV replicon replication. Note that these results are in line with those obtained in previous studies on mammalian cells. Taken together, our findings further support the CHIKV replicon-mediated eGFP expression and suggest that this proposed replicon system could be utilized for the evaluation of antiviral treatments.

### Evaluating the functionality of CHIKV replicon variants in dual-host cells

**Figure 6:**
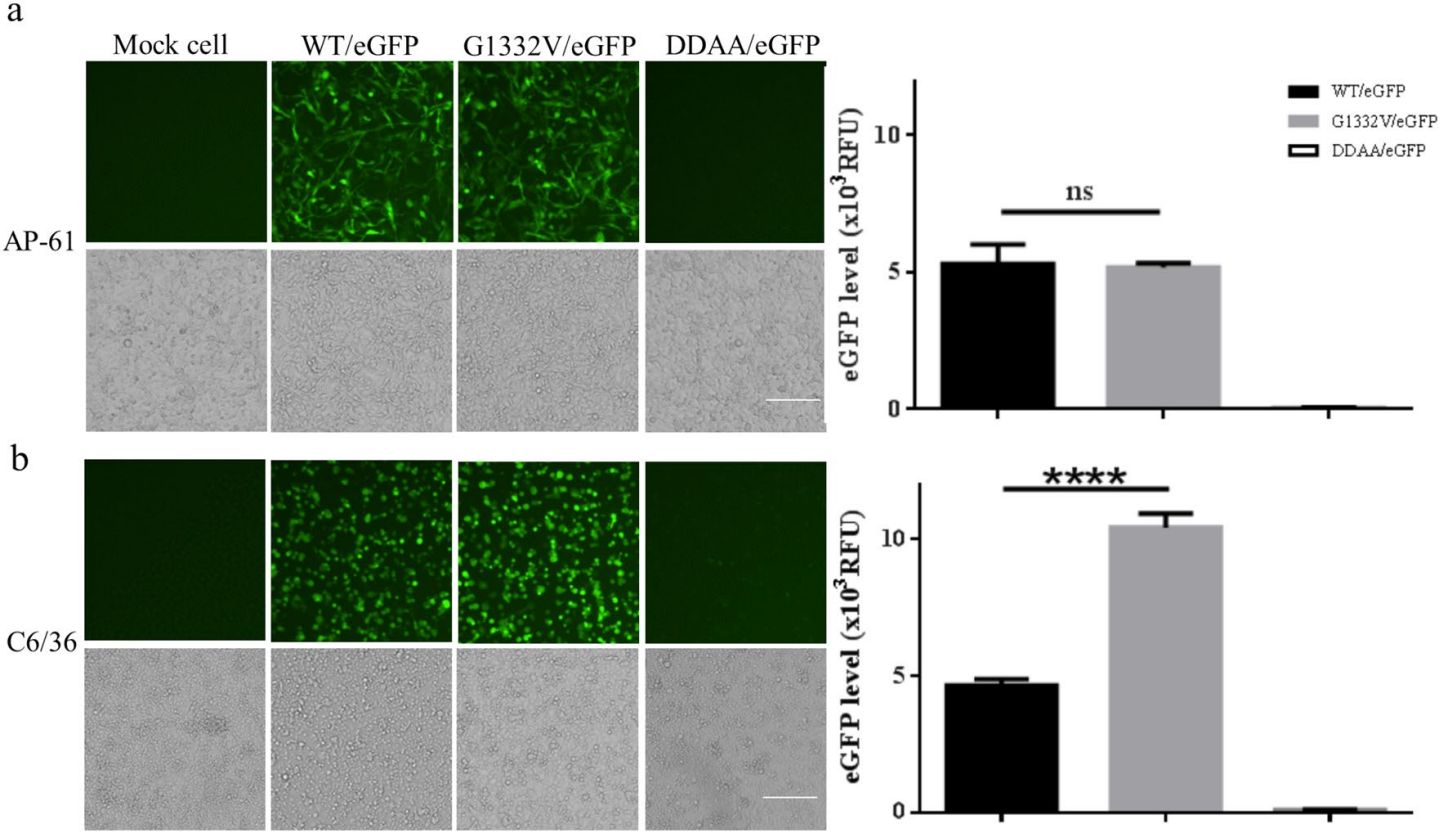
Autonomous CHIKV replicon-mediated eGFP expression of replicon variants in mosquito cell lines. AP-61 (**a**) or C6/36 (**b**) cells were transduced in triplicate using either WT/eGFP, G1332V/eGFP, or DDAA/eGFP at indicated MOIs of 2. At 2 dpt, eGFP-expressing cells were photographed using green (upper panels) or bright field (lower panels) filters. Expression levels of eGFP were quantified (right part). Error bars represent the standard deviation. The statistical significance between WT/eGFP and G1332V/eGFP was analyzed using the Student’s t-test **(**ns, not significant and ∗∗∗∗*p* < 0.0001).

**Figure 7:**
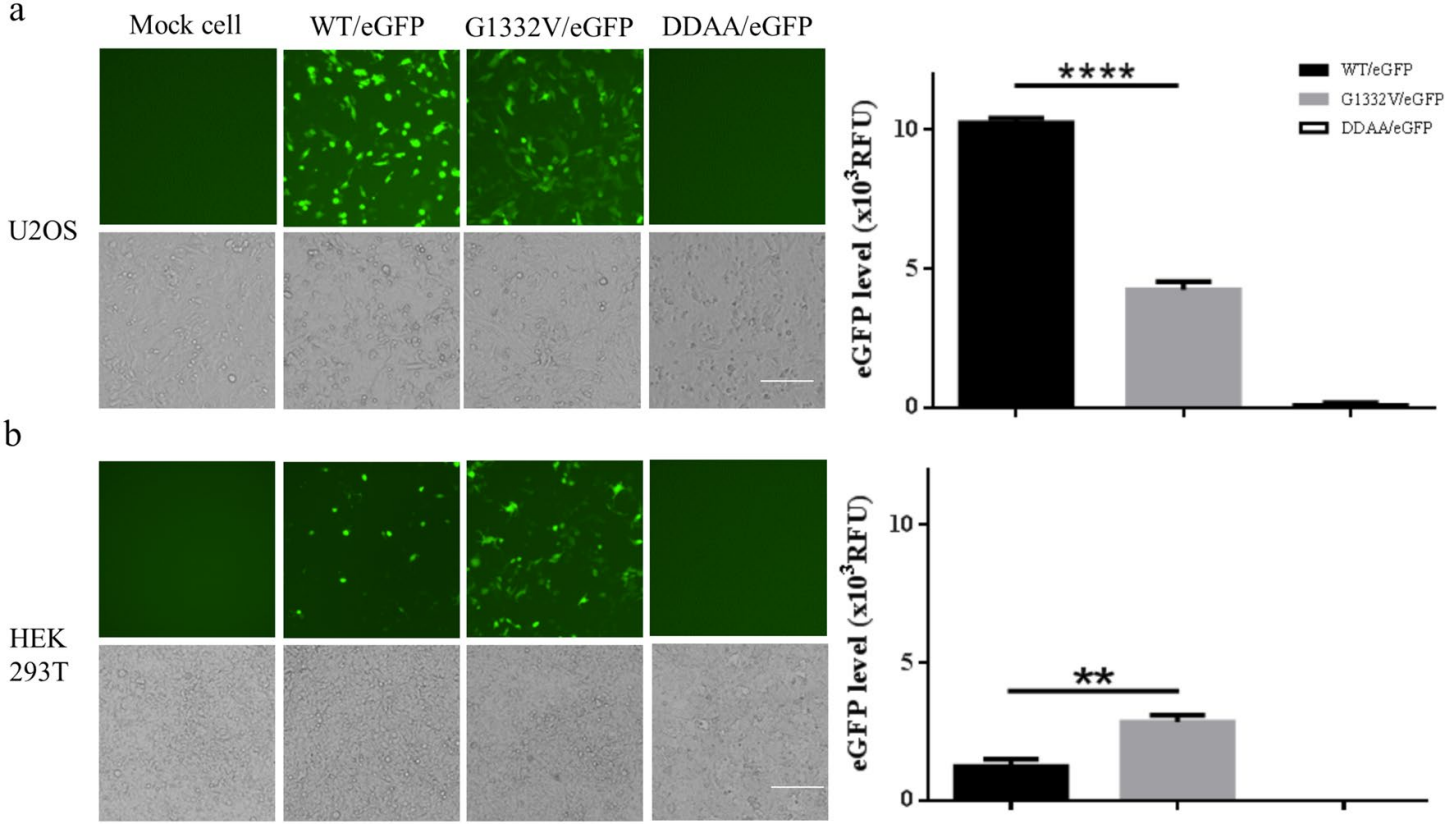
Autonomous CHIKV replicon-mediated eGFP expression of replicon variants in mammalian cell lines. U2OS (**a**) or HEK293T (**b**) cells were transduced in triplicate using either WT/eGFP, G1332V/eGFP, or DDAA/eGFP at an indicated MOI of 10. At 2 dpt, eGFP-expressing cells were photographed using green (upper panels) or bright field (lower panels) filters. Expression levels of eGFP were quantified (right part). Error bars represent the standard deviation. The statistical significance between WT/eGFP and G1332V/eGFP was analyzed using a Student’s t-test (**p < 0.01 and ∗∗∗∗*p* < 0.0001).

**Figure 8:**
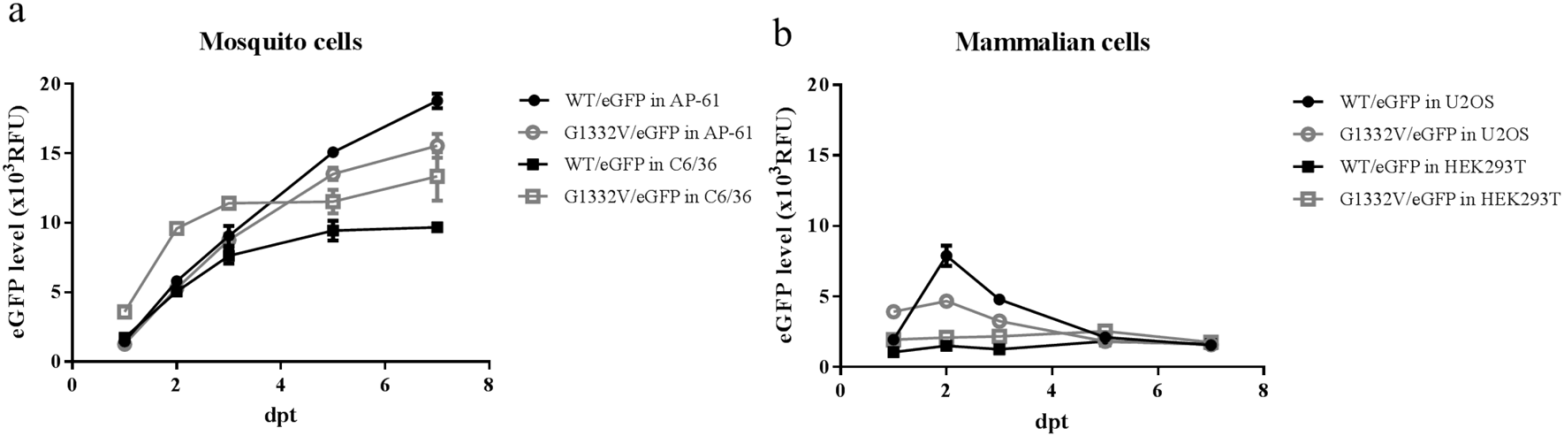
Time course of CHIKV replicon-mediated eGFP expression of replicon variants in mosquito cell lines (**a**, AP-61 and C6/36) or mammalian cell lines (**b**, U2OS and HEK293T) following transduction in triplicate using a recombinant baculovirus at an MOI of 2 or 10, respectively. Expression levels of eGFP were quantified at 1, 2, 3, 5, and 7 dpt. Error bars represent the standard deviation.

**Figure 9:**
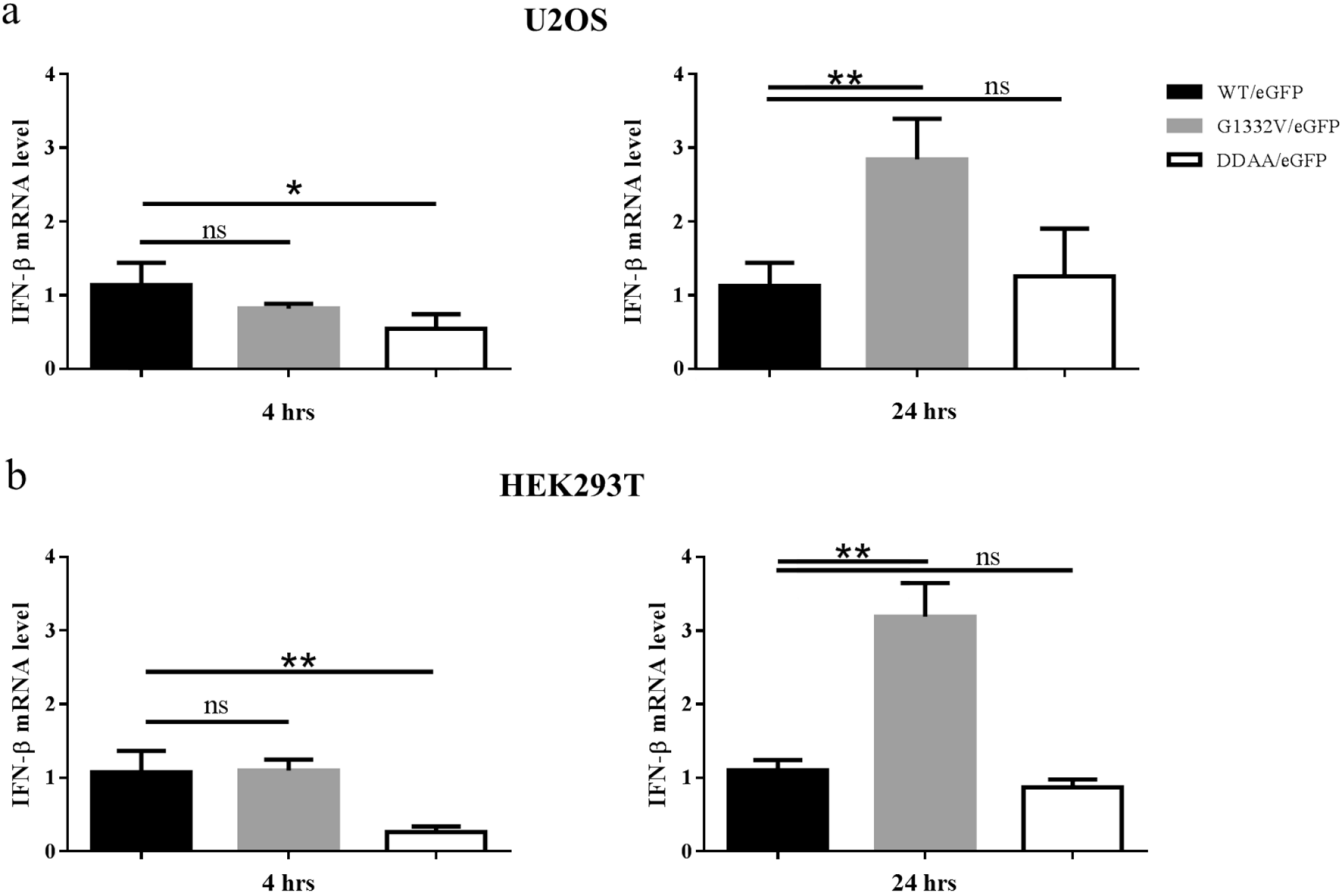
Induction of human IFN-β mRNA by CHIKV replicon variants. U2OS (**a**) or HEK293T (**b**) cells were transduced in triplicate using either WT/eGFP, G1332V/eGFP, or DDAA/eGFP at an MOI of 10. After incubation for 4 h (left panels) or 24 h (right panels), total RNA was harvested and subjected to qRT-PCR for quantification of IFN-β mRNA expression and normalized to HPRT. Error bars represent the standard deviation. Statistical significance was analyzed using Student’s t-test (ns, not significant; *p < 0.05 and **p < 0.01).

To evaluate the functionality of CHIKV replicon for eGFP expression, recombinant baculoviruses bearing either a null functional DDAA mutation or enhanced G1332V mutation (Fig. 1b) were constructed. CHIKV replicon-launched eGFP expression levels of WT/eGFP, G1332V/eGFP, and DDAA/eGFP were examined in mosquito and mammalian cell lines. As shown in Figs. 6 and 7, eGFP signals were detected in all G1332V/eGFP-transduced dual-host cells at 2 days post transduction (dpt). Interestingly, eGFP intensity in G1332V/eGFP-transduced HEK293T and C6/36 cells was significantly higher than WT/eGFP transduced those both cells. By contrast, the expression level of eGFP of WT/eGFP was extremely significant higher than eGFP level of G1332V/eGFP in U2OS cells. The eGFP intensity of G1332V/eGFP was lower than that of WT/eGFP at 5 and 7 dpt in AP-61 cells (Fig. 8a). As expected, no eGFP signal was observed in either DDAA/eGFP-transduced mosquito and mammalian cell lines, which indicates that eGFP expression can be attributed to the functional CHIKV replicon. In time-course assays (Fig. 8), we observed the accumulation of intense eGFP signals in all transduced mosquito cells throughout incubation from 1 to 7 dpt. Note that in transduced mammalian cells, eGFP signals of peak intensity were observed at 2 dpt. Levels of eGFP expression were higher in transduced mosquito cells (10–20 × 10^3^ RFU) than in transduced mammalian cells (< 10 × 10^3^ RFU) at 7 dpt. To determine whether the pattern of CHIKV replicon-launched eGFP expression was associated with innate immunity status, we measured the level of IFN-β mRNA in transduced human cells (U2OS and HEK293T) at 4 and 24 post-transduction (hpt) using qRT-PCR. As shown in Fig. 9, at 4 hpt, IFN-β mRNA levels in WT/eGFP- and G1332V/eGFP-transduced human cells were significantly higher than in DDAA/eGFP-transduced human cells; however, we did not observe a statistically significant difference between WT/eGFP- and G1332V/eGFP-transduced human cells. At 24 hpt, IFN-β mRNA levels in G1332V/eGFP-transduced U2OS and HEK293T cells were significantly higher than in WT/eGFP transduced cells; however, we did not observe a statistically significant difference between WT/eGFP- and DDAA/eGFP-transduced human cells. This indicates that G1332V/eGFP is associated with the potency of IFN-β mRNA induction. Taken together, these results demonstrate the efficacy of the proposed replicon system in characterizing CHIKV replicon variants in terms of replicon-launched eGFP expression (replication) and the potency of IFN-β induction.

### Functional analysis of in vivo delivery system into mosquitos

**Figure 10:**
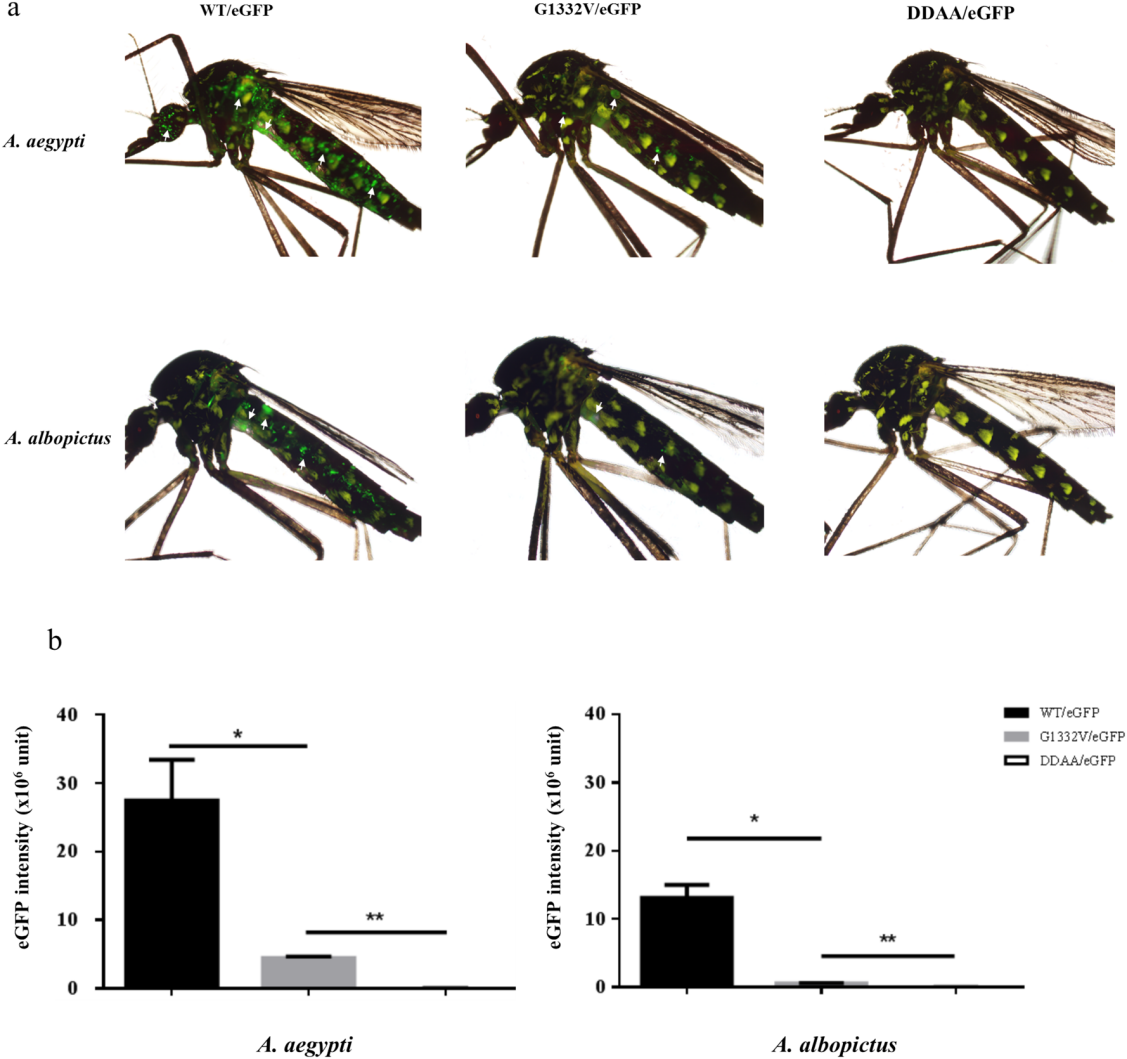
In vivo functional analysis of CHIKV replicon variants in mosquitoes. Adults of *A. aegypti* (upper panels) and *A. albopictus* (lower panels) were intrathoracically injected with either WT/eGFP (left panels), G1332V/eGFP (middle panels), or DDAA/eGFP (right panels) at a dose of 5 × 10^6^ pfu/mosquito. The eGFP expression (indicated by white arrows) was photographed (**a**) and quantified (**b**). Statistical significance was analyzed using Student’s t-test (*p < 0.05 and **p < 0.01).

The efficacy of the proposed in vivo delivery system was evaluated by intrathorically injecting WT/eGFP, G1332V/eGFP, or DDAA/eGFP into adult mosquitoes of two species (*A. aegypti* and *A. albopictus*). Those eGFP signals were observed in both species of mosquito following injection with WT/eGFP or G1332V/eGFP. Time course results (data not shown) revealed that eGFP signals can be attributed to gene turn-on rather than carry over. As shown in Fig. 10a, the eGFP signals at 5 dpt were more pronounced in WT/eGFP-injected mosquitoes than in G1332V/eGFP-injected mosquitoes. Of note, the eGFP signals were more pronounced in *A. aegypti* mosquitoes than in *A. albopictus* mosquitoes. As expectedly, no eGFP signals were detected in DDAA/eGFP-injected mosquitoes. Quantitative results (Fig. 10b) of eGFP intensity confirm that levels of eGFP signal in WT/eGFP-injected both mosquitoes were significantly higher than eGFP level in corresponding G1332V/eGFP-injected mosquitoes. These results demonstrate the efficacy of the proposed system in the in vivo delivery of functional CHIKV replicons into adult mosquito hosts.

## Discussion

Reverse genetics is an important tool in the elucidation of viral replication and the development of novel vaccine strategies^22,30–32^. Note however that nearly all reverse genetics systems are based on the cells of vertebrates, despite the fact that the lifecycle of arboviruses involves mosquitoes as well as mammals (i.e., invertebrates and vertebrates). Thus, a simple universal method by which to introduce genomes or replicons into both mammalian and mosquito cells could facilitate the study of functional genomics pertaining to arboviruses as well as the prediction of outbreaks and the development of countermeasures. Viral vectors that are accessible to mammalian cells in vitro may provide an efficient system for the autonomous delivery of replicons into target cells^24,33,34^; however, no such system has been reported specifically for mosquito cells. Furthermore, most reverse genetics systems designed for positive-sense single-stranded RNA viruses associated with the *Aedes* mosquito cell line are based on the transfection or electroporation of DNA- and RNA-based replicons. Note that the efficiency of the RNA delivery systems is very low (0.2–10%)^27^. One study reported an improved transfection protocol to overcome these technological issues for insect cells^28^. Efforts to use reverse genetics for arboviruses will require a new approach to the delivery of replicons into mosquitoes and mammalian cells.

In the current study, we developed a baculovirus-mediated CHIKV replicon delivery system (Fig. 1), in which a CHIKV cDNA replicon is delivered into mosquitoes and mammalian cells via transduction under the control of a CMV promoter. The use of a single baculovirus vector eliminates the trouble and expense of capped IVT-RNA preparation and electroporation. Baculovirus/CHIKV replicon-launched eGFP expression was validated via co-localization among an eGFP reporter, nsP1, and dsRNA (Fig. 4) as well as inhibition via 6-AU treatment (Fig. 5) or the introduction of a DDAA mutation in RdRp (Figs. 6, 7). CHIKV replicon-launched eGFP expression was more pronounced in mosquito cells than in mammalian cells (Figs. 2, 3, 8), due perhaps to a lower rate of transduction in mammalian cells^29^. eGFP expression was more pronounced in transduced-U2OS cells than in transduced-HEK293T cells, due largely to the efficiency of baculovirus transduction^35^. Alphavirus nsP2 virulence can be characterized in terms of the effect on the host antiviral response via two down-regulatory pathways: (i) IFN-β transcription via global host shutoff and (ii) JAK-STAT signaling via inhibition of STAT1 phosphorylation and/or nuclear translocation^13,15,36–41^. Interestingly, this study demonstrated that the G1332V mutant (nsP2) is associated with an increase in eGFP expression in innate immunity-deficient C6/36 (from 1 to 7 dpt) and HEK293T cells (at 2 and 3 dpt)^42,43^ and a decrease in eGFP expression in AP-61 (at 5 and 7 dpt) and U2OS cells (at 2 and 3 dpt) (Fig. 6, 7, 8). This is consistent with previous reports that the replication/transcription of CHIKV G1332V mutant did not alter activity in mammalian cells, but induced a 60-fold increase in C6/36 cells^12^. Researchers have reported a variety of defects in the innate immune responses to adenovirus infection in U2OS cells^44^. However, RNA viral infections tend to upregulate IFN-β expression in U2OS cells^45^. The nsP2 G1332V mutant is a potent inducer of IFN-β rapidly activated ISGs in U2OS cells, resulting in the inhibition of CHIKV replicon-launched eGFP expression. G1332V/eGFP-transduced cells (C6/36 and HEK293T) presented a significant increase in replication (eGFP expression), due perhaps to the delayed cleavage of nsP2-nsP3^9^. In C6/36 cells (incompetent innate immunity), the restriction of innate immunity could be relieved resulting in eGFP expression levels of G1332V/eGFP higher than WT/eGFP. HEK293T cells retained their transcriptional pro-inflammatory response but lost their ability to secrete type 1 IFN^43^. In HEK293T cells, G1332V/eGFP transduction also induced high eGFP expression as well as IFN-β mRNA expression, albeit without a concomitant increase in secretion. These findings could imply the conservation of unknown evolutionary mechanisms in mosquito and mammalian cells. Previous studies have reported that point mutations in S-adenosyl-l-methionine (SAM)-dependent RNA methyltransferase-like (SAM MTase-like) domain impair the ability of nsP2 to induce transcriptional shutoff^36^. Likewise, CHIKV G1332V point mutations located in the SAM MTase-like domain could have a deleterious effect on the ability of nsP2 to induce transcriptional shutoff. Another nsP2 mutant (P726G) in SINV also resulted in elevated IFN-β production despite limited virus propagation in vivo^13^. Functional nsP2 in WT/eGFP was shown to induce global transcriptional shutoff via RPB1 degradation^38^, which prevented activation of the transcription-dependent IFN-β response and JAK-STAT signaling and also induced cell death. Conversely, the nsP2 G1332V mutant without the above functionalities was shown to strongly induce IFN-β expression. In cases of SINV infection, the G1332V mutant associated nonfunctional nsP2 strongly promoted α/β interferon production by preventing infected cells from downregulating cellular transcription^46,47^. Note however that replication of the SINV G1332V mutant was defective in mosquito cells but not in vertebrate cells^46^. It appears that nsP2 VLoop mutants are incapable of interfering with the induction of type I interferon attenuated chikungunya virus replication in vitro and in vivo^14^. The nsP1/nsP2/ nsP3 cleavage domains play a critical role in regulating cellular antiviral response induced by the Ross River Virus or Sindbis virus^46–49^. However, the role of the nsP1/nsP2/ nsP3 cleavage domains of CHIKV has yet to be elucidated. In the current study, we demonstrated that the nsP2/ nsP3 cleavage domain drives the CHIKV-induced IFN-β response.

In CHIKV-eGFP based assays, the IC_50_ value for 6-AU was approximately 1 μg/ml (4 μM) (Fig. 5a), which is in good agreement with previous reports on reporter-based stable CHIKV replicon cell lines and CHIKV replication in Vero cells^23,50^. Note that 6-AU did not affect eGFP expression in mosquito cells. Previous studies reported that 6-AU exhibits a moderate anti-arboviral activity in mammalian cells^51,52^ but not in mosquito cells^53^, indicating fundamental differences in the host-CHIKV or drug-host interactions. The transient replicon system developed in the current study could be a valuable tool by which to perform high-throughput antiviral screening, optimize antiviral activities, and elucidate antiviral mechanisms.

To the best of our knowledge, this is the first study to report the in vivo delivery of functional CHIKV replicons into adult mosquitoes (Fig. 10). Following injection with WT/eGFP or G1332V/eGFP, eGFP expression was more pronounced in *A. aegypti* than in *A. albopictus* (Fig. 10a), due to differences in vector competence and/or CMV activity. Those eGFP signals in WT/eGFP-injected both mosquitoes were significantly higher than eGFP level in corresponding G1332V/eGFP-injected mosquitoes (Fig. 10b), due perhaps to the functional nsP2 restriction of innate mosquito immunity to WT CHIKV replication. Note that we have also conducted in vivo assays using another reporter of a near-infrared fluorescent protein (iRFP682) in mice; however, the results to date have been unsuccessful, due perhaps to the sensitivity of baculovirus and/or the restriction of innate immunity^54^. Efforts to elevate MOI and thereby permit the detection of RFP682 expression in mice are currently on-going.

Alphavirus replicons have previously been used to induce heterologous protein expression in mammalian cells^55,56^. This is the first study to demonstrate the use of a baculovirus for the efficient delivery of autonomous CHIKV replicons into mammalian and mosquito cells in vitro as well as adult mosquitoes in vivo*.* The peak CHIKV replicon-launched eGFP expression levels were observed at 2 dpt in mammalian cells and 7 dpt in mosquito cells. Persistent replicon-launched eGFP expression exceeding 30 days (data not shown) highlights the potential of the proposed replicon system for heterologous protein expression using mosquito cells. Previous study has demonstrated that baculovirus does not replicate in mosquito cell line^29^. The continuous increase in eGFP expression in transduced mosquito cells likely results in persistency of CHIKV replicon. Additionally, combining baculovirus/CHIKV systems may make it possible to create a single-round infectious virus from dual-host cells as an alternate approach to vaccine development. The proposed scheme allows the efficient delivery of replicons into mosquito and mammalian cells using a single vector to facilitate analysis of CHIKV replication and the development of treatments. The proposed replicon system was shown to improve the utility of alphavirus expression systems, particularly in mosquito cells. Due to inconsistencies in the persistent infectivity of alphaviruses in mosquitoes and mammalian cells, the proposed system could conceivably be used to develop a new generation of systems for alphavirus replicon-based protein expression in mosquito cells. This system also opens the door to the development of alphavirus VRP packages based on mosquito cells, while facilitating the in vivo delivery of alphavirus replicons into mosquitoes.

## Methods

### Cells and viruses

Cells from mosquito cell line AP-61 (*Aedes pseudoscutellaris*) were cultured in Leibovitz L-15 medium (Gibco) with 10% fetal bovine serum (FBS) (Gibco) and 1% antibiotic–antimycotic (Gibco) at 28 °C. Cells from the mosquito cell line C6/36 (*Aedes albopictus*) (ATCC^®^ CRL-1660™) were cultured in RPMI 1640 Medium (Gibco) with 1% penicillin/streptomycin (Gibco) and 10% FBS (Gibco) at 28 °C under 5% CO_2_. U2OS cells (ATCC HTB-96), HEK293T cells (ATCC^®^ CRL-1573™) cells grown in Dulbecco’s modified Eagle medium (DMEM) (GIBCO) containing 1% GlutaMAX™ (Gibco), 1% penicillin/streptomycin (Gibco) and 10% FBS (Gibco) at 37 °C in 5% CO2. BHK-21 cells (ATCC^®^ CCL-10™) were cultured in Minimum Essential Media (MEM)(Gibco) containing 1% GlutaMAX™(Gibco), 1% MEM NEAA (Gibco), 1% Sodium Pyruvate (Gibco), 1% penicillin/streptomycin (Gibco) and 10% FBS (Gibco) at 37 °C in 5% CO2.

### Generation of recombinant baculoviruses for CHIKV replicons

In constructing a CHIKV replicon transfer vector, we first cloned the glycoprotein gene of Vesicular stomatitis virus (VSVG) (GenBank: J02428.1), which was amplified via PCR using the viral DNA of CellLightTM Early Endosomes-RFP (Invitrogen, Carlsbad, CA, USA) as a template with a pair of primers (forward: 5′-TTGGGATCCTTGACACTATGAAGTGCC-3′; reverse: 5′-AGGGCATGCGTTTAAACCTCGAGGCGATCGCGCAGGATTTGAGTTACTTTCC-3′) inserted into the *Bam*HI and *Sph*I sites of the pFastBac1 vector (Invitrogen, Carlsbad, CA, USA) to create the plasmid, pFastBac1-VSVG-*Sgf*I-*Pme*I. A synthetic 9.5-kb CHIKV replicon-GFP DNA fragment derived from CHIKV strain LR2006_OPY1 (GenBank: KT449801) with structural gene (26S) substitution and green fluorescent protein (EGFP) also subcloned into the *Sgf*I and *Pme*I sites of pFastBac1-VSVG. The resulting plasmid is hereafter referred to as the pFastBac1-VSVG-CHIKV replicon-GFP of the CHIKV replicon transfer vector. Both CHIKV replicon transfer vectors of GAA and G1332V mutants were created by site-directed mutagenesis using PCR based on the sequence of pFastBac1-VSVG-CHIKV replicon-GFP. In constructing the GAA mutant, two PCR fragments of 4.9-kb (forward: 5′-GCCCTACCACGAATTCGCATATGAAGG-3′; reverse: 5′-CCATGTATTATGTTGGCGGCGCCGATGAAGGCC-3′) and 0.4-kb (forward: 5′-GGCCTTCATCGGCGCCGCCAACATAATACATGG-3′; reverse: GCAAAATAGGTAGCGGCCGCTGTAGTGC-3′) were assembled with a large fragment of pFastBac1-VSVG-CHIKV replicon-GFP *Eco*RI-*Not*I using the NEBuilder HiFi DNA Assembly. In constructing the G332V mutant, two PCR fragments of 1.9-kb (forward: 5′-GCCCTACCACGAATTCGCATATGAAGG-3′; reverse: 5′-GGTGCACATACTGCTCGG-3′) and 3.4-kb (forward: 5′-CCGAGCAGTATGTGCACC-3′; reverse: GCAAAATAGGTAGCGGCCGCTGTAGTGC-3′) were assembled with a large fragment of pFastBac1-VSVG-CHIKV replicon-GFP *Eco*RI*-Not*I using the NEBuilder HiFi DNA Assembly. The transfer vectors of both mutants were confirmed by DNA sequencing via PCR. The generation of recombinant baculoviruses for CHIKV replicon was performed in accordance with the protocol of the Bac to Bac expression system (Invitrogen, Carlsbad, CA, USA). Viral titers were determined using the BacPAK™ Baculovirus Rapid Titer Kit (Takara Bio USA, Inc) in accordance with the manufacturer’s instructions.

### Bacoluvirus transduction

Mosquito or mammalian cells were seeded in 24-well plates (2 × 10^5^ cells/well or 1 × 10^5^ cells/well) and in 96-well plates (2 × 10^4^ cells/well or 1 × 10^4^ cells/well). On the following day, cells were transduced with baculovirus at an indicated multiplicity of infection (MOI) in phosphate-buffered saline, pH7.4 (PBS) (Gibco) at 28 °C or 37 °C for 1 h (hr). Cells were washed using PBS once prior to the addition of growth culture medium. The cells were cultured for various durations as indicated.

### IFA

In generating rabbit anti-nsP1 serum, a 911-bp *Nco*I*–Sma*I fragment (AF369024, 637–1548) of partial nsp1 gene from the pFastBac1-VSVG-CHIKV replicon-GFP was subcloned into the restriction enzyme sites of pET32c. The expression, purification, and rabbit immunization of recombinant nsp1 were performed in accordance with methods described in a previous report^57^. AP-61 cells were transduced using WT/eGFP at an MOI of 5. At 4 dpt, cells were fixed and incubated using the above-mentioned rabbit anti-nsp1 serum (1:100) or Mab anti-GFP antibodies (Santa Cruz Biotechnology, Inc.) (1:100) and mouse anti-dsRNA antibody (SCICONS) for 1 h. Cells were subsequently washed three times using PBS and incubated with either Alexa Fluor 594-conjugated secondary antibodies or Alexa Fluor 488-conjugated secondary antibodies as well as Hoechst 33342 (10 µM) for 1 h. After a final wash using PBS, images of cells were captured using an inverted fluorescence microscope (DMi8 Microscope, Leica Microsystems).

### eGFP quantification

Cells in triplicate collected from 24-well plates were lysed using 200 µL/well RIPA buffer (Thermo Scientific™). The eGFP expression was measured using DS-11 FX + spectrophotometer/fluorometer (DeNovix, USA) in accordance with the manufacturer’s instructions. Three independent experiments in time course assay were performed with very similar results, and the data presented represent the results from one independent experiment.

### Inhibition assay

U2OS and AP-61 cells were seeded in 24-well plates at a density of 1 × 10^5^ cells/well. On the following day, U2OS cells were transduced using either WT/eGFP or CMV-GFP (CellLight™ Early Endosomes-GFP, Invitrogen) as a negative control, respectively at on MOI of 5 or 1. AP-61 cells were transduced using WT/eGFP at an MOI of 1. Transduced cells were cultured in triplicate using growth medium in the presence 6-AU (Sigma-Aldrich) at various indicated concentrations. Following incubation for 24 h, cells were subjected to eGFP quantification assay. In cell viability assays, U2OS or AP-61 cells were seeded in 96-well plate at a density of 2 × 10^4^ cells/well. On the following day, cells were cultured in triplicate using growth medium in the presence 6-AU at various concentrations for 24 h. Cell viability was determined using the Cell Counting Kit-8 kit (Enzo) in accordance with the manufacturer’s instructions. Absorbance at 450 nm was measured using a microplate reader Infinite^®^ 200 PRO (Tecan Trading, Ltd., Switzerland). Cell viability was evaluated in terms of absorbance at various doses divided by the absorbance at 0 ug/ml. Three independent experiments were performed with very similar results, and the data presented represent the results from one independent experiment.

### Quantitative analysis of IFN-β mRNA

U2OS or HEK293T cells (24-well plate) were transduced with the indicated recombinant baculovirus at an MOI of 10. Total RNA was harvested at 4 or 24 hpt using a Monarch^®^ Total RNA Miniprep Kit (NEB). One-step, quantitative RT–PCR was performed using the one-step reverse transcriptase PCR (RT-PCR) kit (Qiagen) in a total volume of 25 μL. qRT–PCR was conducted using the ABI StepOnePluses Real Time PCR System (Applied Biosystems) in triplicate. The primer sequences used in this assay were as follows: HPRT-F, 5′-ATC AGA CTG AAG AGC TAT TGT AAT GA-3′; HPRT-R, 5′-TGG CTT ATA TCC AAC ACT TCG TG-3′; IFN-b-F, 5′-GTC TCC TCC AAA TTG CTC TC-3′; and IFN-b-R, 5′-ACA GGA GCT TCT GACACT GA-3′^45^. Assessment of IFN-β expression was based on relative quantification (RQ) using the comparative critical threshold (CT) value method. IFN-β expression was normalized to hypoxanthine–guanine phosphoribosyltransferase (HGPRT) encoded in humans by the endogenous control gene HPRT1. Fold changes were calculated using the ddCt algorithm based on WT/eGFP samples as a reference. Two independent experiments were performed with very similar results, and the data presented represent the results from one independent experiment.

### Mosquito transduction in vivo

Adult *A. aegypti* (Kaoshiung strain) and *A. albopictus* (Chung-Ho strain) mosquitoes were reared at 27 °C under 80% humidity with a 12-h light/dark cycle. Cold-anesthetized mosquitoes were intra-thoracically inoculated with indicated viruses at a dose of 5 × 10^6^ plaque forming units (pfu)/mosquito. At 5 dpt, cold-anesthetized mosquitoes were photographed using an IX71 inverted fluorescence microscopy (Olympus). The levels of eGFP intensity of images was quantitated with Image J 1.53e version. We narrowed the hue tone of the color threshold (54 to 112) for the specific intensity of green color. The methods of quantification are outlined in the Image J documentation: ‘‘Image → Adjust → Color Threshold → Measure’’. The eGFP intensity was defined as (Area X Mean fluorescence of readings). For calculation of relative intensities, the pixel intensity was normalized with the fluorescent signal of PBS-injected mosquitoes. Two mosquitoes were analyzed for each genotype.

### Statistical analysis

Data were analyzed using GraphPad Prism 6.01 software. Data was assessed for statistically significant differences using a two-tailed, unpaired t-test. A *p* value < 0.05 was considered statistically significant. *p* values were indicated as follows: ns, not significant; *p < 0.05, significant; **p < 0.01, highly significant; ∗∗∗*p* < 0.001 and ∗∗∗∗*p* < 0.0001, extremely significant.

## Supplementary information

Supplementary Information.

## References

[1] Strauss JH, Strauss EG (1994). The alphaviruses: gene expression, replication, and evolution. Microbiol. Rev..

[2] Silva LA, Dermody TS (2017). Chikungunya virus: epidemiology, replication, disease mechanisms, and prospective intervention strategies. J. Clin. Investig..

[3] Powers AM, Logue CH (2007). Changing patterns of chikungunya virus: re-emergence of a zoonotic arbovirus. J. Gen. Virol..

[4] Higgs S, Vanlandingham D (2015). Chikungunya virus and its mosquito vectors. Vector Borne Zoonotic Dis. Larchmont NY.

[5] Weaver SC, Lecuit M (2015). Chikungunya virus and the global spread of a mosquito-borne disease. N. Engl. J. Med..

[6] Staples JE, Breiman RF, Powers AM (2009). Chikungunya fever: an epidemiological review of a re-emerging infectious disease. Clin. Infect Dis..

[7] Khan AH (2002). Complete nucleotide sequence of chikungunya virus and evidence for an internal polyadenylation site. J. Gen. Virol..

[8] Solignat M, Gay B, Higgs S, Briant L, Devaux C (2009). Replication cycle of chikungunya: a re-emerging arbovirus. Virology.

[9] Rupp JC, Sokoloski KJ, Gebhart NN, Hardy RW (2015). Alphavirus RNA synthesis and non-structural protein functions. J. Gen. Virol..

[10] Li YG (2013). Chikungunya virus induces a more moderate cytopathic effect in mosquito cells than in mammalian cells. Intervirology.

[11] Fros JJ, Pijlman GP (2016). Alphavirus infection: host cell shut-off and inhibition of antiviral responses. Viruses.

[12] Bartholomeeusen K (2018). A chikungunya virus trans-replicase system reveals the importance of delayed nonstructural polyprotein processing for efficient replication complex formation in mosquito cells. J. Virol..

[13] Frolova EI (2002). Roles of nonstructural protein nsP2 and Alpha/Beta interferons in determining the outcome of Sindbis virus infection. J. Virol..

[14] Meshram CD (2019). Lack of nsP2-specific nuclear functions attenuates chikungunya virus replication both in vitro and in vivo. Virology.

[15] Fros JJ, van der Maten E, Vlak JM, Pijlman GP (2013). The C-terminal domain of chikungunya virus nsP2 independently governs viral RNA replication, cytopathicity, and inhibition of interferon signaling. J. Virol..

[16] Göertz GP (2018). The methyltransferase-like domain of chikungunya virus nsP2 inhibits the interferon response by promoting the nuclear export of STAT1. J. Virol..

[17] Dubensky TW (1996). Sindbis virus DNA-based expression vectors: utility for in vitro and in vivo gene transfer. J. Virol..

[18] Xiong C (1989). Sindbis virus: an efficient, broad host range vector for gene expression in animal cells. Science.

[19] Goertz GP (2018). Conserved motifs in the hypervariable domain of chikungunya virus nsP3 required for transmission by *Aedes aegypti* mosquitoes. PLoS Negl. Trop. Dis..

[20] Gao Y, Goonawardane N, Ward J, Tuplin A, Harris M (2019). Multiple roles of the non-structural protein 3 (nsP3) alphavirus unique domain (AUD) during chikungunya virus genome replication and transcription. PLoS Pathog..

[21] Ohlund P (2018). DNA-launched RNA replicon vaccines induce potent anti-Ebolavirus immune responses that can be further improved by a recombinant MVA boost. Sci. Rep..

[22] Lundstrom K (2019). Plasmid DNA-based alphavirus vaccines. Vaccines.

[23] Pohjala L (2011). Inhibitors of alphavirus entry and replication identified with a stable chikungunya replicon cell line and virus-based assays. PLoS ONE.

[24] Vasilakis N (2003). Transfection-independent production of alphavirus replicon particles based on poxvirus expression vectors. Nat. Biotechnol..

[25] Pan Y (2009). Efficient gene delivery into mammalian cells by recombinant baculovirus containing a hybrid cytomegalovirus promoter/Semliki Forest virus replicon. J. Gene Med..

[26] Guan M (2006). Increased efficacy and safety in the treatment of experimental liver cancer with a novel adenovirus–alphavirus hybrid vector. Cancer Res..

[27] Boylan BT, Moreira FR, Carlson TW, Bernard KA (2017). Mosquito cell-derived West Nile virus replicon particles mimic arbovirus inoculum and have reduced spread in mice. PLoS Negl. Trop. Dis..

[28] Atieh T (2017). New reverse genetics and transfection methods to rescue arboviruses in mosquito cells. Sci. Rep..

[29] Naik NG (2018). Baculovirus as an efficient vector for gene delivery into mosquitoes. Sci. Rep..

[30] Aubry F, Nougairède A, Gould EA, de Lamballerie X (2015). Flavivirus reverse genetic systems, construction techniques and applications: a historical perspective. Antiviral Res..

[31] Komdeur FL (2020). First-in-human phase I clinical trial of an SFV-based RNA replicon cancer vaccine against HPV-induced cancers. Mol. Ther. J. Am. Soc. Gene Ther..

[32] Singh A, Koutsoumpli G, van de Wall S, Daemen T (2019). An alphavirus-based therapeutic cancer vaccine: from design to clinical trial. Cancer Immunol. Immunother. CII.

[33] Sánchez-Puig JM, Lorenzo MM, Blasco R (2013). A vaccinia virus recombinant transcribing an alphavirus replicon and expressing alphavirus structural proteins leads to packaging of alphavirus infectious single cycle particles. PLoS ONE.

[34] Sun Y (2011). A novel alphavirus replicon-vectored vaccine delivered by adenovirus induces sterile immunity against classical swine fever. Vaccine.

[35] Wang CH, Naik NG, Liao LL, Wei SC, Chao YC (2017). Global screening of antiviral genes that suppress baculovirus transgene expression in mammalian cells. Mol. Ther. Methods Clin. Dev..

[36] Akhrymuk I, Lukash T, Frolov I, Frolova EI (2019). Novel mutations in nsP2 abolish chikungunya virus-induced transcriptional shutoff and make the virus less cytopathic without affecting its replication rates. J. Virol..

[37] Simmons JD, Wollish AC, Heise MT (2010). A determinant of Sindbis virus neurovirulence enables efficient disruption of Jak/STAT signaling. J. Virol..

[38] Akhrymuk I, Kulemzin SV, Frolova EI (2012). Evasion of the innate immune response: the Old World alphavirus nsP2 protein induces rapid degradation of Rpb1, a catalytic subunit of RNA polymerase II. J. Virol..

[39] Breakwell L (2007). Semliki Forest virus nonstructural protein 2 is involved in suppression of the type I interferon response. J. Virol..

[40] Fros JJ (2010). Chikungunya virus nonstructural protein 2 inhibits type I/II interferon-stimulated JAK-STAT signaling. J. Virol..

[41] Simmons JD (2009). Venezuelan equine encephalitis virus disrupts STAT1 signaling by distinct mechanisms independent of host shutoff. J. Virol..

[42] Brackney DE (2010). C6/36 Aedes albopictus cells have a dysfunctional antiviral RNA interference response. PLoS Negl. Trop. Dis..

[43] Ferreira CB (2020). Lentiviral vector production titer is not limited in HEK293T by induced intracellular innate immunity. Mol. Ther. Methods Clin. Dev..

[44] Laredj LN, Beard P (2011). Adeno-associated virus activates an innate immune response in normal human cells but not in osteosarcoma cells. J. Virol..

[45] Thompson MR (2014). Interferon γ-inducible protein (IFI) 16 transcriptionally regulates type I interferons and other interferon-stimulated genes and controls the interferon response to both DNA and RNA viruses. J. Biol. Chem..

[46] Gorchakov R (2008). A new role for ns polyprotein cleavage in Sindbis virus replication. J. Virol..

[47] Gorchakov R, Frolova E, Frolov I (2005). Inhibition of transcription and translation in Sindbis virus-infected cells. J. Virol..

[48] Liu X (2018). Decreased virulence of Ross River virus harboring a mutation in the first cleavage site of nonstructural polyprotein is caused by a novel mechanism leading to increased production of interferon-inducing RNAs. MBio.

[49] Cruz CC (2010). Modulation of type I IFN induction by a virulence determinant within the alphavirus nsP1 protein. Virology.

[50] Briolant S, Garin D, Scaramozzino N, Jouan A, Crance JM (2004). In vitro inhibition of Chikungunya and Semliki Forest viruses replication by antiviral compounds: synergistic effect of interferon-alpha and ribavirin combination. Antiviral Res..

[51] Pascoalino BS, Courtemanche G, Cordeiro MT, Gil LH, Freitas-Junior L (2016). Zika antiviral chemotherapy: identification of drugs and promising starting points for drug discovery from an FDA-approved library. F1000Research.

[52] Crance JM, Scaramozzino N, Jouan A, Garin D (2003). Interferon, ribavirin, 6-azauridine and glycyrrhizin: antiviral compounds active against pathogenic flaviviruses. Antiviral Res..

[53] Dong S, Kang S, Dimopoulos G (2019). Identification of anti-flaviviral drugs with mosquitocidal and anti-Zika virus activity in *Aedes aegypti*. PLoS Negl. Trop. Dis..

[54] Hofmann C, Strauss M (1998). Baculovirus-mediated gene transfer in the presence of human serum or blood facilitated by inhibition of the complement system. Gene Ther..

[55] DiCiommo DP, Bremner R (1998). Rapid, high level protein production using DNA-based Semliki Forest virus vectors. J. Biol. Chem..

[56] Liljestrom P, Garoff H (1991). A new generation of animal cell expression vectors based on the Semliki Forest virus replicon. Biotechnology (N. Y).

[57] Kuo SC (2011). Cell-based analysis of Chikungunya virus membrane fusion using baculovirus-expression vectors. J. Virol. Methods.

