# Supplementary material for: Facile method for delivering chikungunya viral replicons into mosquitoes and mammalian cells
Source: Sci Rep. 2021 Jun 10;11:12321. doi: 10.1038/s41598-021-91830-y (PMC8192953; doi:10.1038/s41598-021-91830-y)
Supplement: Supplementary file 1 — Supplementary Information. [file 41598_2021_91830_MOESM1_ESM.docx]

**Supplementary Information**

**Facile method for delivering chikungunya viral replicons into mosquitoes and mammalian cells**

Hui-Chung Lin^1,3^, Der-Jiang Chiao^1^, Chang-Chi Lin^1,2^, Szu-Cheng Kuo^1,2,*^

**Supplementary Materials and Methods**

***In vitro* transcribed (IVT)-mRNA and electroporation**

Linearized plasmid DNA of pFastBac1-VSVG-CHIKV replicons encoding eGFP obtained via PmeI (NEB) digestion served as a template. IVT-RNA was produced using T7 RNA polymerase (RiboMAX™ Large Scale RNA Production Systems, Promega). IVT-mRNA was additionally capped using the ScriptCap™ m7G capping system (Cellscript) and purified via phenol-chloroform extraction. Finally, IVT-mRNA was analyzed via agarose gel electrophoresis (Formaldehyde-Free RNA Gel Kits, AMRESCO) and stored at -70 °C. BHK-21 cells were diluted to a density of 1x10^6^ in Gene Pulser Electroporation Buffer (BioRad). A total of 50 μg of IVT-mRNA of WT/eGFP was electroporated into BHK-21 cells using the Gemini X2 Electroporation System (BTX) under the following operating parameters: 300 V, 140 Ω, 3.5ms, 2 pulses. Following incubation for 24 hrs, electroporated BHK-21 cells were visualized using an IX71 inverted fluorescence microscopy (Olympus).

**Supplementary Figure**


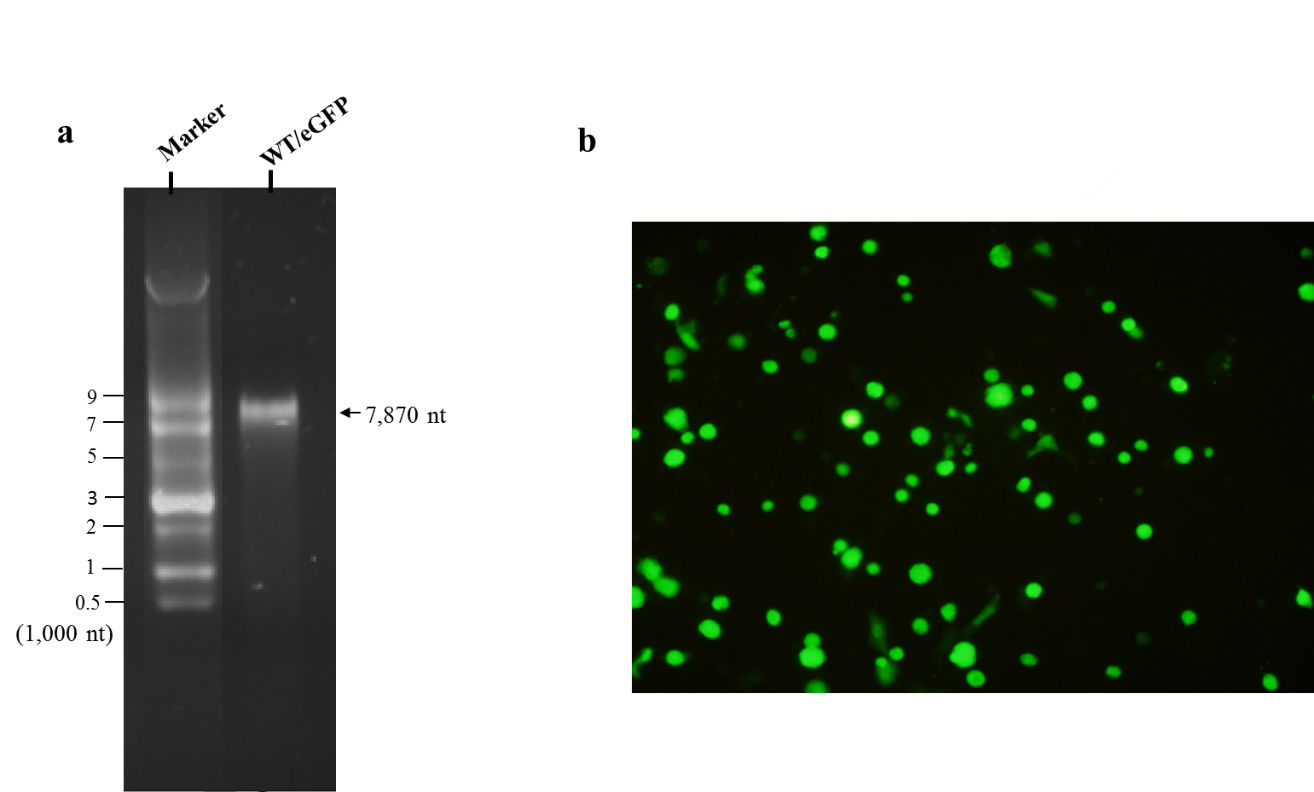


Supplementary Figure 1. Functional analysis of IVT mRNA from a transfer vector of WT/eGFP. (a) Agarose gel electrophoresis of IVT-mRNA. Electrophoresis was performed on a 1% formaldehyde free RNA gel. lane1: ssRNA marker, lane2: IVT-mRNA of WT/eGFP (7,870 nt). The nucleotide (nt) numbers of RNA are indicated on both sides. (b) Analysis of CHIKV replicon-mediated expression of eGFP following IVT-mRNA transfection. A 50 μg IVT mRNA of WT/eGFP electroporated into 1x10^6^ BHK-21 cells. After incubation for 24 hrs, cells were visualized using a green filter.

**Supplementary Material**


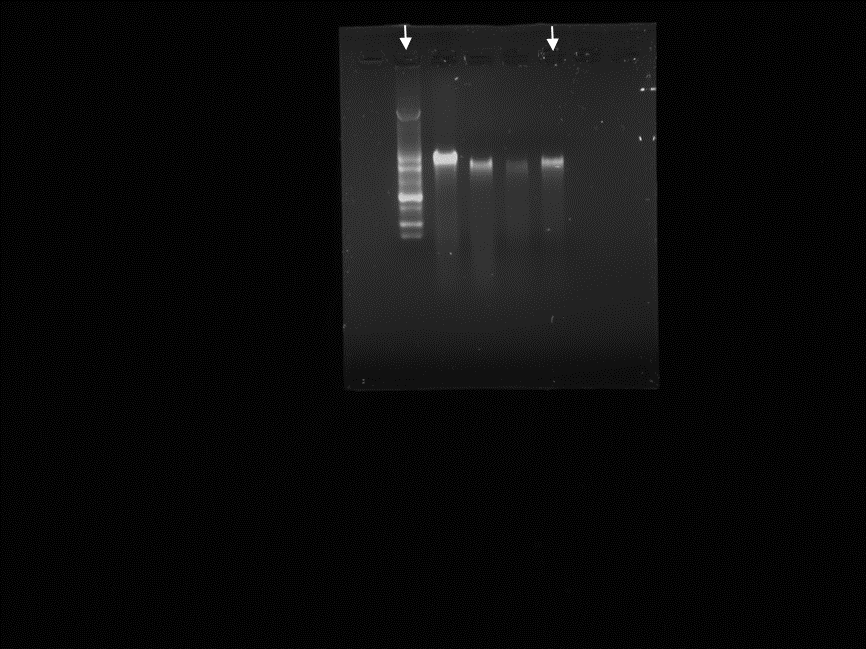
**Supplementary Figure. Full length gel of the agarose gel electrophoresis of IVT-mRNA data shown in the Figure S1. Marker and IVT mRNA of WT/eGFP were indicated by arrows.**
